# Unveiling the role of phages in shaping the periodontal microbial ecosystem

**DOI:** 10.1128/msystems.00201-25

**Published:** 2025-03-28

**Authors:** Fangfang Yao, Jiajun He, Raphael Nyaruaba, Hongping Wei, Yuhong Li

**Affiliations:** 1 State Key Laboratory of Oral & Maxillofacial Reconstruction and Regeneration, Key Laboratory of Oral Biomedicine Ministry of Education, Hubei Key Laboratory of Stomatology, School & Hospital of Stomatology Wuhan University 660005, Wuhan, Hubei, China; 2 CAS-Key Laboratory of Synthetic Biology, CAS Center for Excellence in Molecular Plant Sciences, Institute of Plant Physiology and Ecology, Chinese Academy of Sciences 74519, Shanghai, China; 3 University of Chinese Academy of Sciences, Beijing, China; 4 WHP Innovation Lab, Wuhan Institute of Virology Chinese Academy of Sciences 74614 , Wuhan, Hubei, China; University of Birmingham, Birmingham, United Kingdom

**Keywords:** periodontitis, phage, prophage, phage-bacteria interactions, auxiliary genes, genomic diversity

## Abstract

The oral microbiome comprises various species and plays a crucial role in maintaining the oral ecosystem and host health. Phages are an important component of the periodontal microbiome, yet our understanding of periodontal phages remains limited. Here, we investigated oral periodontal phages using various advanced bioinformatics tools based on genomes of key periodontitis pathogens. Prophages were found to encode various auxiliary genes that potentially enhance host survival and pathogenicity, including genes involved in carbohydrate metabolism, antibiotic resistance, and immune modulation. We observed cross-species transmission among prophages with a complex network of phage-bacteria interactions. Our findings suggest that prophages play a crucial role in shaping the periodontal microbial ecosystem, influencing microbial community dynamics and the progression of periodontitis.

**IMPORTANCE:**

In the context of periodontitis, the ecological dynamics of the microbiome are largely driven by interactions between bacteria and their phages. While the impact of prophages on regulating oral pathogens has been increasingly recognized, their role in modulating periodontal disease remains underexplored. This study reveals that prophages within key periodontitis pathogens contribute significantly to virulence factor dissemination, antibiotic resistance, and host metabolism. By influencing the metabolic capabilities and survival strategies of their bacterial hosts, prophages may act as critical regulators of microbial communities in the oral cavity. Understanding these prophage-mediated interactions is essential not only for unraveling the mechanisms of periodontal disease progression but also for developing innovative therapeutic approaches that target the microbial ecosystem at the genetic level. These insights emphasize the need for more comprehensive studies on the ecological risks posed by prophages in shaping microbial pathogenicity and resistance.

## INTRODUCTION

Periodontitis is a chronic and irreversible inflammatory disease that progressively damages the supporting structures of the teeth, including the gums, periodontal ligament, and alveolar bone (1). Periodontitis arises from a complex interplay between the host immune response and pathogenic microorganisms within the oral cavity (2). The primary initiator of periodontitis is the accumulation of subgingival plaques, which serve as a reservoir of diverse microbial communities. These plaques not only harbor periodontal pathogens, such as *Porphyromonas gingivalis*, *Tannerella forsythia*, *Treponema denticola*, *Prevotella intermedia*, *Fusobacterium nucleatum*, and *Aggregatibacter actinomycetemcomitans* but also contain a significant population of viruses, including phages and other viral entities (3). Understanding the microbial and viral dynamics within subgingival plaques is crucial to unraveling the mechanisms underlying periodontitis progression and developing more effective therapeutic strategies.

Phages, viruses that infect bacteria, are categorized into two types based on their life cycle: strictly lytic phages (or virulent) and temperate phages (4). Temperate phages, upon infecting a bacterial host, integrate their genome into the host’s chromosome, entering a latent state known as a prophage (4, 5). Under favorable environmental conditions, a prophage can reactivate, switching to a lytic phase, during which it undergoes replication and eventually causes the lysis of the host bacterium. Prophages play a crucial role in bacterial processes, providing metabolic advantages or resistance mechanisms that enhance the competitiveness of their host cells within microbial communities (5–8). The majority of viral populations in the oral microbiome are phages (9). Compared to healthy individuals, the composition of phages in the subgingival plaque biofilm of patients with periodontitis exhibits a similar community structure, suggesting that changes in the phage community are associated with periodontitis (10). Further studies have revealed that in the oral microbiome, phage-bacteria interactions form a cross-linked network rather than specific predator-prey relationships, indicating that phages may play a crucial role in either preventing or exacerbating the deterioration of the oral ecosystem (11). In addition, phages have been shown to shape bacterial interactions with the human host immune system, with their encoded proteins participating in oral immune response and disease progression (12, 13). This highlights the role of phages as regulators of oral disease within the human oral microbiome.

Advancements in genomics have enhanced our understanding of the potential roles of phages in the development, regulation, and treatment of pathogenic microbiota in periodontal and other oral sites (14, 15). The impact of phage communities on human periodontal health and disease remains underexplored. Since their discovery over a century ago, phages associated with periodontal pathogens have rarely been isolated, resulting in a lack of cultivable bacteria-phage model systems to elucidate microbial community ecology in the periodontal microbiome (9, 16). Limited studies have investigated the key periodontal pathogen—*P. gingivalis* (17, 18). However, the influence of these prophages on the survival and pathogenic potential of host bacteria remains insufficiently understood.

Understanding prophages not only provides insights into the potential role of phages in periodontal health management but also holds significant importance for studying bacterial ecology from a genomic perspective. This study aims to investigate the impact of integrated prophages on the ecology of key periodontal pathogens, focusing on their contributions to mobile elements, virulence factors, and antibiotic resistance genes in key periodontal pathogens. In addition, we constructed a phage-bacteria interaction network to explore the regulatory role of phages within the oral microbial ecosystem. Our findings reveal the influence of the largely uncharacterized phage community on the periodontal microbiome, underscoring the importance of phages in future studies on periodontal ecology.

## RESULTS

### The features of prophages in the key periodontitis-related species

**Fig 1:**
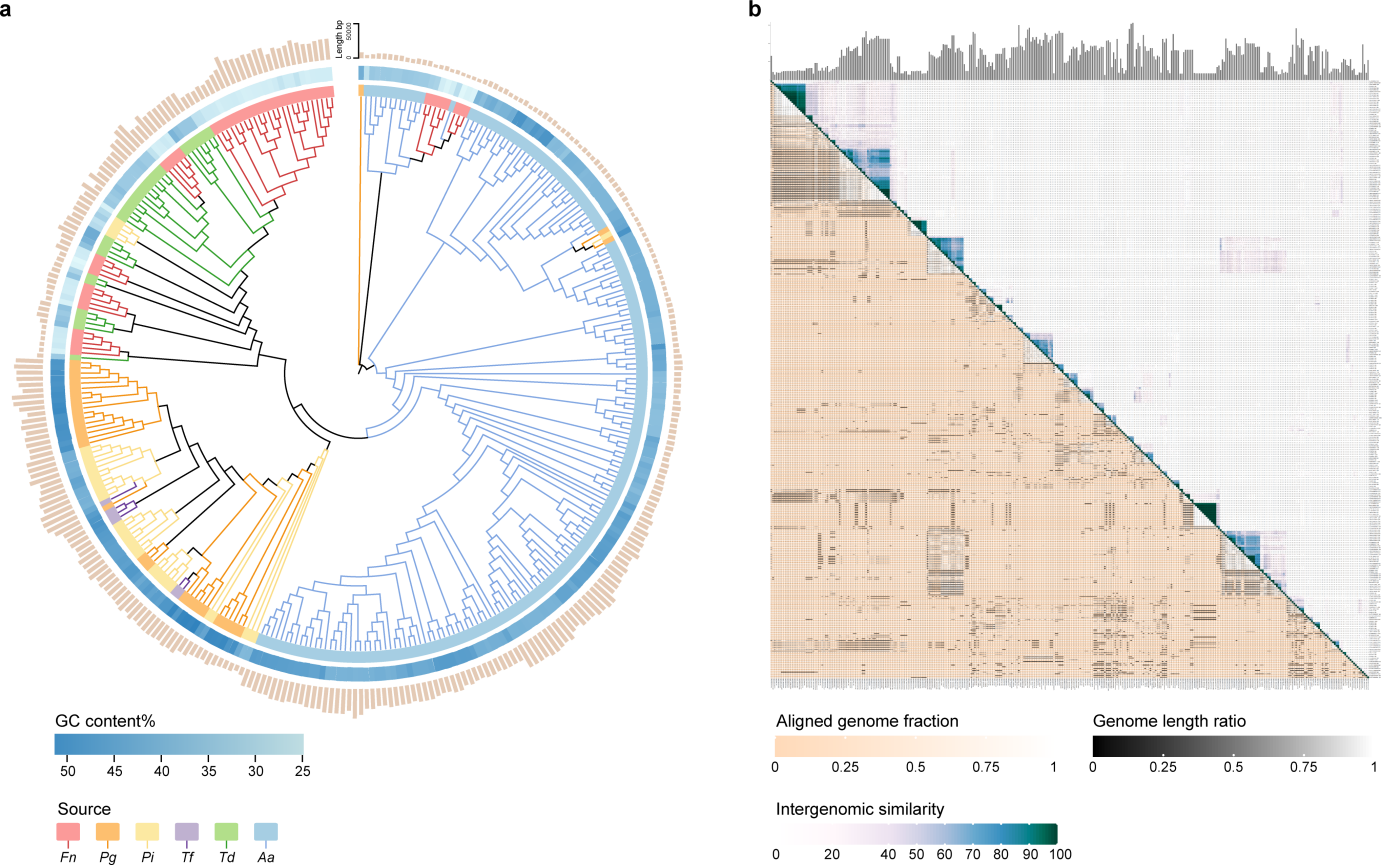
Genome analysis of the key periodontitis pathogens, prophage sequences. (a) A phylogenetic tree of prophage is presented. The different colored blocks inside indicate the host bacteria of the prophage. The abbreviations represent the following bacterial strains: *F. nucleatum* (Fn), *P. gingivalis* (Pg), *P. intermedia* (Pi), *T. forsythia* (Tf), *T. denticola* (Td), and *A. actinomycetemcomitans* (Aa). The blue blocks adjacent to the source circles denote the GC content of the prophages. The outer pale peach bars represent the length of the prophages. (b) Intergenomic similarity analysis of the prophage sequences was performed using VIRIDIC, resulting in a heatmap that displays both intergenomic similarity values (on the right side) and alignment indicators (on the left side and top annotation), darker colors on the right represent higher genome similarity, with numbers indicating similarity values for each genome pair, rounded to one decimal place. On the left, darker shades highlight lower values, signifying genome pairs with either limited alignment coverage (displayed in an orange-to-white gradient) or differences in genome lengths (shown in a black-to-white gradient).

To explore the role of phages in periodontal health and disease, we focused on studying prophages in the key periodontitis pathogens. We systematically screened the genomes of 364 bacterial strains, covering six major periodontal pathogens (*P. gingivalis*, *T. forsythia*, *T. denticola*, *P. intermedia*, *F. nucleatum*, and *A. actinomycetemcomitans*), and 52.5% were identified as carrying at least one prophage (Table S1A and B). To better understand the diversity of prophages in these pathogens, we constructed a phylogenetic tree using all the predicted prophages from our data set (Fig. 1a). Previously reported phages often exhibit narrow host ranges and species specificity, even limited to specific strains (19). Surprisingly, we found prophages from different bacterial species in more than one phylogenetic cluster, indicating that cross-species transmission may occur among phages within periodontal pathogens. Notably, although phages within the same phylogenetic cluster were closely related, their bacterial hosts were not always the most phylogenetically similar. We further analyzed these prophage sequences using VIRIDIC, a tool that employs the standard algorithm from the International Committee on Taxonomy of Viruses (ICTV) and the Bacterial and Archaeal Viruses Subcommittee to measure viral genome similarity (20). The heatmap generated by VIRIDIC integrated the genomic similarity values of these prophages with information about genome length and aligned genome fraction (Fig. 1b). Only a small portion of these prophages exhibited high similarity (in dark blue), while the majority showed very low similarity to each other (in white). These findings suggest that the prophages in the genomes of the key periodontal pathogens exhibit high genetic diversity.

### Genomic characteristics of prophages in the key periodontitis-related species genomes

**Fig 2:**
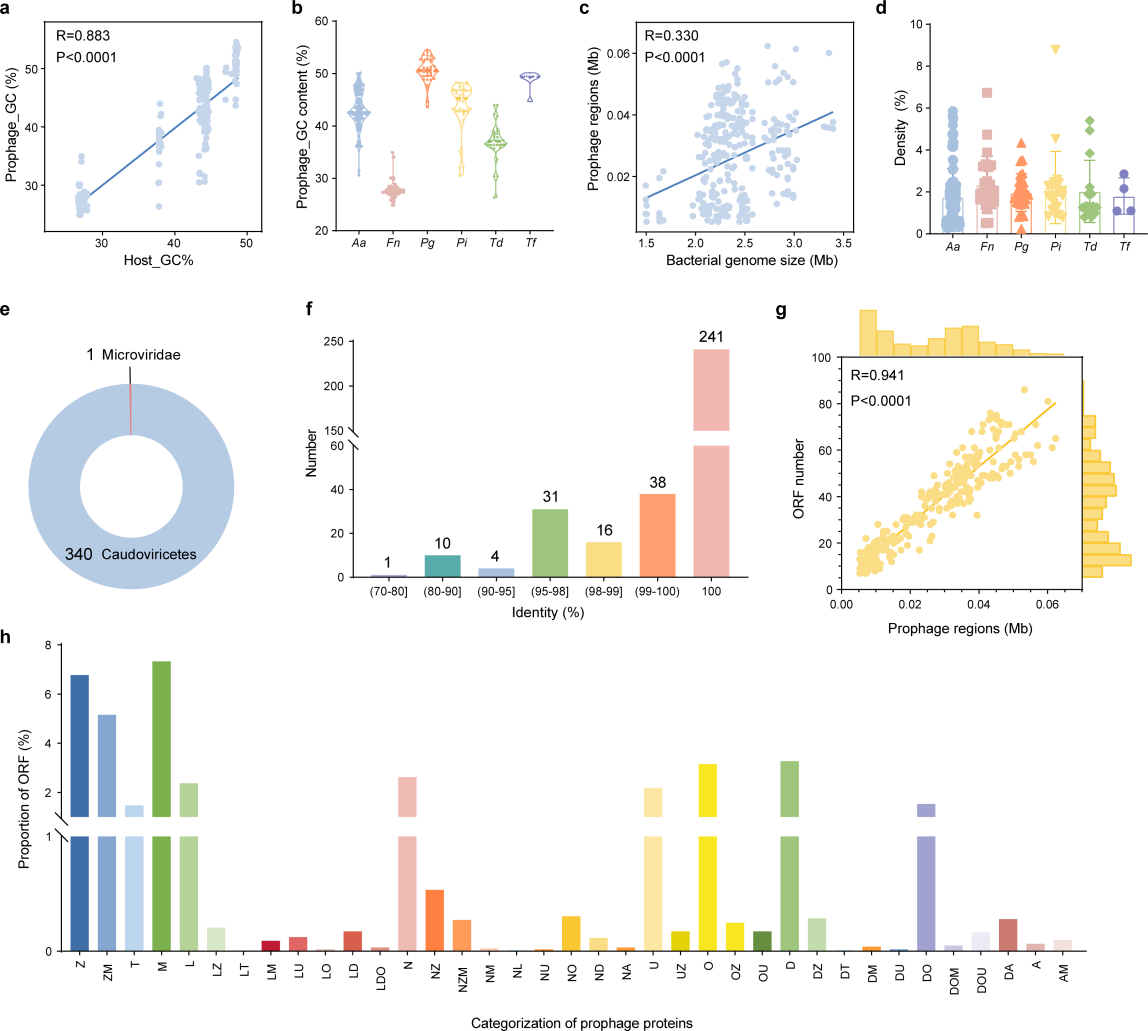
General genome features of prophages in the key periodontitis pathogens genome. (a) Scatter plot showing the relationship of the GC content between prophages and host. (b) GC_content% of prophages from different host bacteria. (c) Correlation between genome size of host and prophage regions. (d) The density of prophages per host genome is relative to the size of the bacterial genome (Mb). (e) Taxonomic annotation and distribution of the prophages. (f) The novelty of prophage sequences compared to viral genomes in the IMG/VR databases. (g) Number of ORFs carried in prophage genome. (h) The proportion of ORFs assigned to each function category for prophage. Functional annotations were determined using PhageScore. Data for function categories of “unsorted” and “hypothetical” are not shown. Function categories are as follows: Assembly (Z), Assembly/Infection (ZM), Immune (T), Infection (M), Integration (L), Integration/Assembly (LZ), Integration/Immune (LT), Integration/Infection (LM), Integration/Packaging (LU), Integration/Regulation (LO), Integration/Replication (LD), Integration/Replication/Regulation (LDO), Lysis (N), Lysis/Assembly (NZ), Lysis/Assembly/Infection (NZM), Lysis/Infection (NM), Lysis/Integration (NL), Lysis/Packaging (NU), Lysis/Regulation (NO), Lysis/Replication (ND), Lysis/tRNA (NA), Packaging (U), Packaging/Assembly (UZ), Regulation (O), Regulation/Assembly (OZ), Regulation/Packaging (OU), Replication (D), Replication/Assembly (DZ), Replication/Immune (DT), Replication/Infection (DM), Replication/Packaging (DU), Replication/Regulation (DO), Replication/Regulation/Infection (DOM), Replication/Regulation/Packaging (DOU), Replication/tRNA (DA), tRNA (A), tRNA/infection (AM).

To further understand the characteristics of prophages in the key periodontitis pathogens, we conducted an in-depth analysis of their genomes. The guanine and cytosine content (GC content) of the prophages in these pathogens ranged from 24% to 55%, showing a significant positive correlation with the GC content of their hosts (Fig. 1a and 2a). We observed apparent differences in the GC content among prophages from different hosts (Fig. 2b). The average genome size of phages was 25.93 kb, exhibiting a weak correlation with the size of the host genome (Fig. 2c). This observed correlation can likely be attributed to the inherent limitations imposed by the bacterial genome size on the capacity to harbor mobile genetic elements (MGEs). Specifically, bacterial species with larger genomes possess a greater capacity to accommodate larger and more complex genetic elements, including phages (21–24). In addition, the mean phage genome density was 2.02% and there were notable differences in prophage content among different bacterial strains. For instance, in one strain of *P. intermedia*, prophages accounted for 8.78% of the total gene content, whereas in other strains, it was less than 1% (Fig. 2d).

To further characterize the prophages, PhageScope was used to determine their taxonomic identities. Apart from one phage classified as Microviridae, the rest were identified as Caudoviricetes (Fig. 2e). Caudoviricetes is a dominant order of prophages in the human oral cavity, with a broad host range spanning multiple phyla (16, 25–27). Then the sequences of prophages were compared to the viral genomes in the Integrated Microbial Genomes/Viral Database (IMG/VR), which is the largest collection of cultured and uncultured viruses (28). The result showed that one prophage from *A. actinomycetemcomitans* showed only 73.08% sequence similarity to the virus in IMG/VR (Fig. 2f). This suggests that these prophages represent novel, uncharacterized groups with potential for further investigation.

To understand the potential effect of these prophages on key periodontal pathogens, we used various advanced bioinformatics tools to annotate the functions of prophage genes. First, we systematically identified and classified the open reading frames (ORFs) in the prophage genomes. We found a significant positive correlation between prophage genome size and the number of ORFs (Fig. 2g). Analysis of the ORFs in these prophage genomes revealed that the majority of prophage-encoded genes (60.55%) could not be easily annotated and were classified as “unknown function.” Among the annotated phage genes, most were core phage proteins involved in phage structure, replication, and lysis (Fig. 2h). Interestingly, some phage ORFs may play important roles in bacterial metabolism and virulence, which we then characterized further.

### Prophages encode various putative auxiliary genes linked with horizontal gene transfer

**Fig 3:**
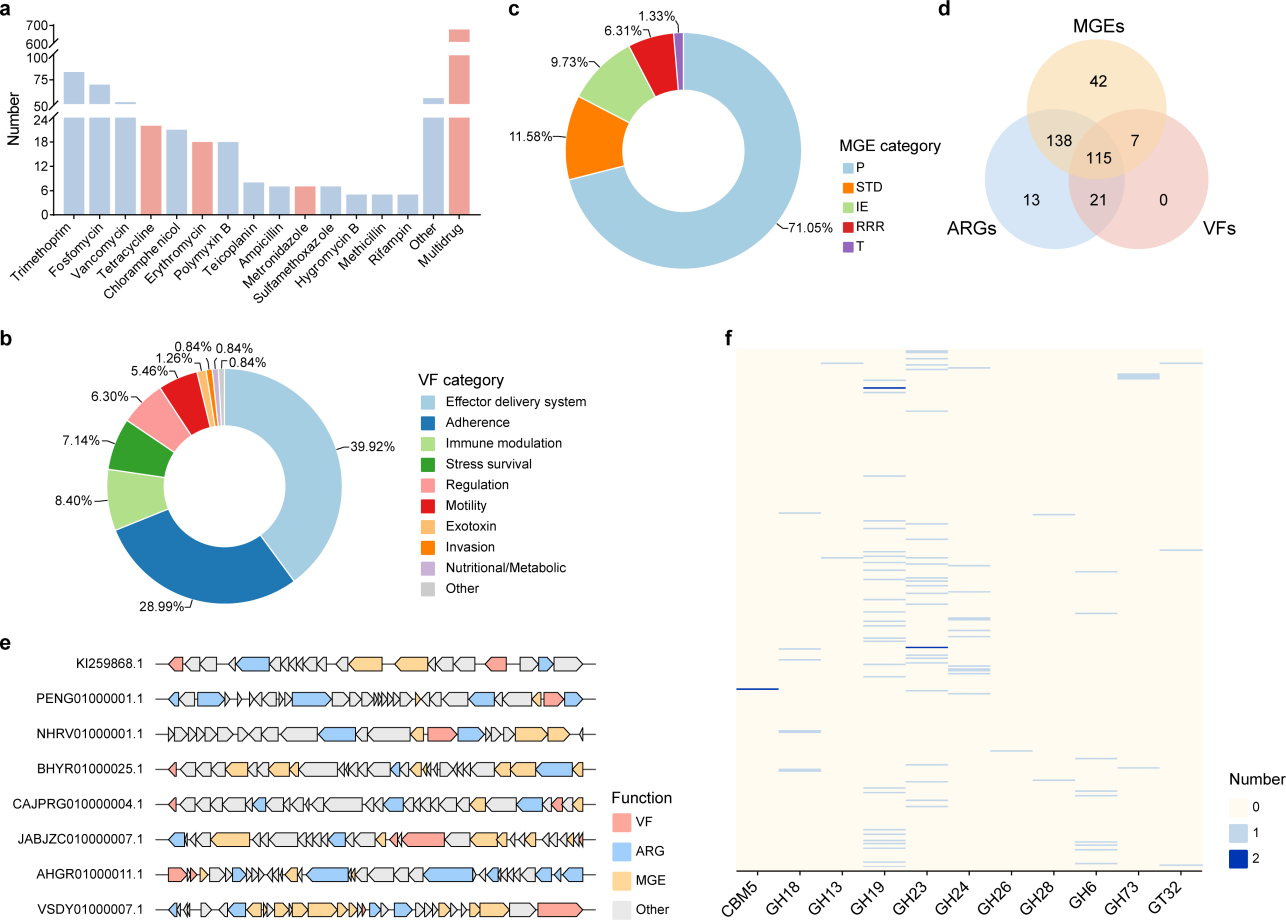
Prophages encode putative auxiliary genes. (a) The stacked bar plot shows the counts for the antimicrobial resistance types identified. Red bars indicate multidrug resistance genes and resistance genes associated with antibiotics commonly used in periodontitis treatment. (b) The proportion of virulence factor (VF) types in prophage. (c) The proportion of MGEs in prophage. MGE categories are as follows: Integration and excision (IE) from one genetic locus to another; Replication, recombination, or nucleic acid repair (RRR); Interorganism transfer (T); Element stability, transfer, or defense (STD); and Phage (P). (d) Venn diagram illustrating the number of unique and shared genes from prophages. (e) Local genome maps of representative prophages containing VF, ARG, and MGE. Genes were marked by different colors to illustrate VF genes (red), ARGs (blue), MGEs (orange), and other phage genes (grey). (f) Heatmap analysis of CAZymes. The frequencies of CAZymes are marked by different colors.

Minocycline is currently widely used to treat periodontitis, but genes encoding tetracycline resistance are the most common among oral bacteria (29, 30). To determine whether prophages in the main periodontal pathogens harbor resistance genes, we utilized the Comprehensive Antibiotic Resistance Database (CARD) to identify and extract all antimicrobial resistance genes (ARGs) from prophage regions and analyze their associated antibiotics. Results indicated that among the 341 prophages, 287 carried antibiotic resistance genes, which were associated with various classes of antibiotics (Fig. 3a). Of these, tetracyclines, metronidazole, and erythromycin are the most commonly used antibiotics in the clinical treatment of periodontitis (31, 32) (Fig. 3a, red bar), which poses a potential challenge for effective treatment. This indicates that key periodontitis pathogens’ prophage-encoded resistance genes may act as vectors for the transfer of resistance genes, posing a serious threat to human health.

Next, we utilized the Virulence Factor Database (VFDB) to identify putative virulence factors (VFs) within the prophages. We performed BLAST similarity searches for the ORFs encoded by the prophages against the VFDB. A total of 238 VFs were predicted, which are involved in regulation, adhesion, stress survival, immune modulation, effector delivery systems, and motility (Fig. 3b). Among them, the EF-Tu gene related to adhesion was the most abundant. The EF-Tu exposed on the bacterial surface can interact with oral cell receptors, promoting the invasion and colonization of pathogens (33, 34). Following this, the exeA gene, associated with the effector delivery system, was the next most prevalent, which is essential for the secretion of many virulence factors, such as aerolysin, proteases, and hemolysin (35, 36). In addition, the genes encoded by prophages also included virulence-related genes linked to proteases, iron (III) ABC transporters, oligopeptide transport, and flagellar motility. This suggests that prophages are involved in building the virulence arsenal of the periodontitis pathogens.

Horizontal gene transfer (HGT) is a process that promotes gene circulation within ecological microbial communities, in which phages play a crucial role by mediating transduction events that enable host bacteria to acquire genes enhancing virulence, antibiotic resistance, and other traits. To investigate how these prophages drive horizontal gene transfer, we further identified the MGEs within the prophages using mobileOG-db. As expected, in addition to MGEs associated with phage-specific biological processes, there are a large number of other types of MGEs in prophages, such as Integration and excision (IE) from one genetic locus to another; Replication, recombination, or nucleic acid repair (RRR); Interorganism transfer (T); Element stability, transfer, or defense (STD) (Fig. 3c). Interestingly, most phages harboring VFs or ARGs also encode proteins involved in MGEs (Fig. 3d). A large number of MGEs were found in the gene clusters of VF or ARGs and their genomic neighborhoods (the five genes upstream or downstream) (Fig. 3e; Fig. S1). This result suggests that VFs and ARGs may spread widely among bacterial populations, enhancing the pathogenicity and resistance of the periodontal microbiome and altering the genetic diversity of the host.

Considering the potential involvement of phages in carbohydrate metabolism within the periodontal ecosystem, we further annotated the genes encoded by phages according to the carbohydrate-active enzyme (CAZymes) database (dbCAN3). A total of 98 CAZymes were identified in the prophages, including 93 glycoside hydrolases (GHs), 3 glycosyltransferases (GTs), and 2 carbohydrate-binding modules (CBMs) (Fig. 3f). The presence of these CAZymes may enhance the host bacteria’s ability to utilize carbon sources, providing specific adaptive and competitive advantages to the host bacteria within the ecosystem.

### Prophages encode various auxiliary metabolic genes to modulate bacterial host metabolism

**Fig 4:**
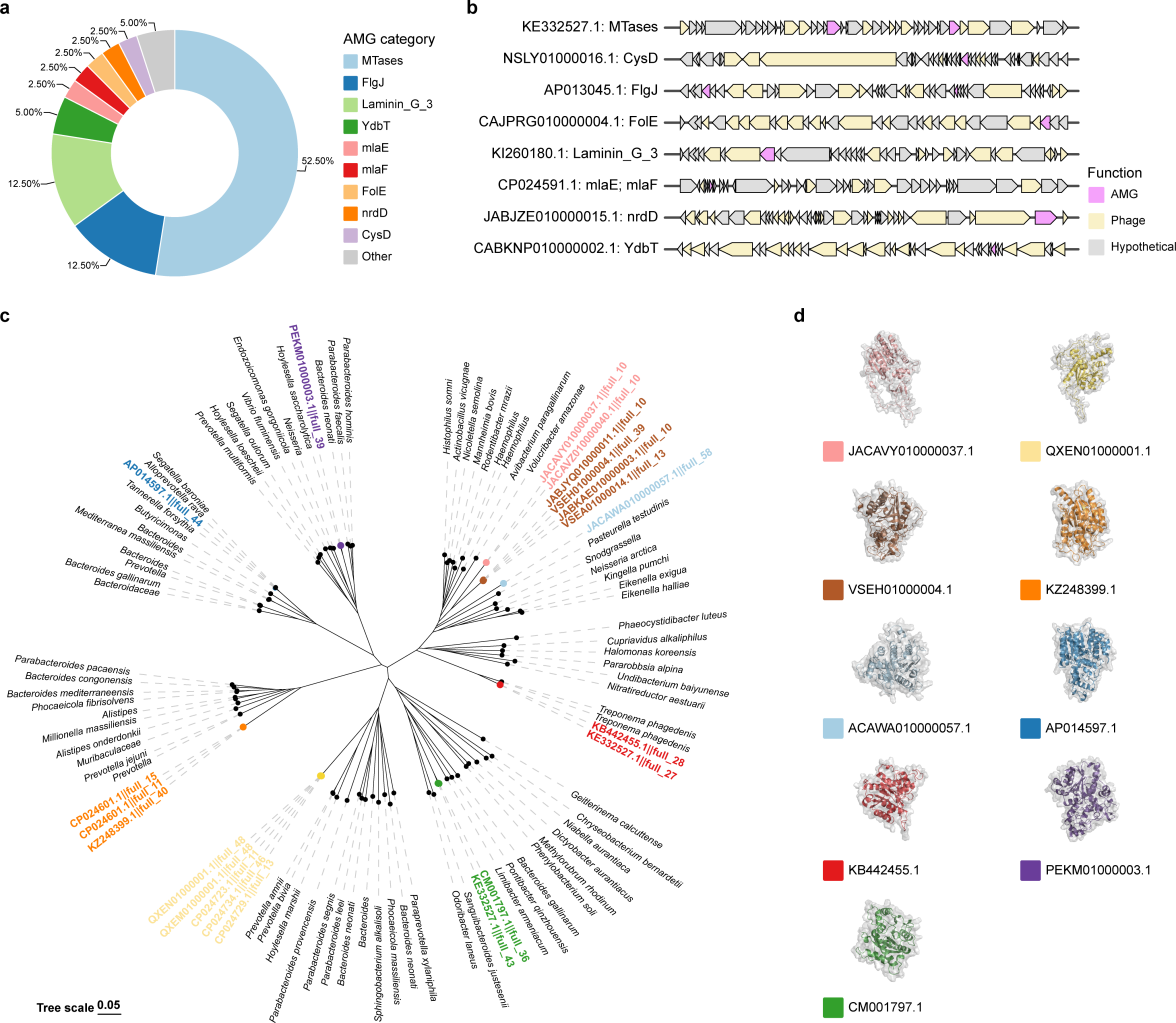
Characterization of auxiliary metabolic genes in prophages. (a) Distribution of auxiliary metabolic genes across prophages. (b) Local genome maps of representative prophages carrying the auxiliary metabolic genes. Genes were marked by different colors to AMGs (cerise), phage protein genes (yellow), and hypothetical protein genes (gray). (c) A phylogenetic tree illustrating the viral and microbial MTases genes, with viral sequences indicated in color and microbial sequences in black. (d) Predicted structures of representative MTase proteins in prophage.

As previously reported in various ecosystems, phages can also encode host genes to drive host metabolism (37, 38). To identify putative auxiliary metabolic genes (AMGs) that might hijack host metabolism, we used DRAM-v to predict AMGs within the key periodontitis pathogens prophage. We identified typical AMGs in prophages, involved in nucleotide metabolism, phospholipid metabolism, motility, and adhesion, including genes like DNA (cytosine-5-)-methyltransferase (MTases), ribonucleoside-triphosphate reductase (nrdD), phospholipid/cholesterol/gamma-HCH transport system permease protein (mlaE; mlaF), FolE, CysD, Laminin_G_3, and FlgJ (Fig. 4a and b; Fig. S2 and S3).

We observed that certain bacterial hosts harbor prophages carrying specific AMGs, which may play distinct roles in bacterial survival (Fig. S2). Among the AMGs, MTases were the most common MTase activity that is crucial for regulating bacterial cellular functions, and these phages might influence bacterial metabolism through epigenetic modification via autonomous DNA methylation (39–41). Phylogenetic analysis of MTases in the prophages and MTases from the NCBI RefSeq database suggested that phages might have acquired the genes from bacteria (Fig. 4C). The structures of representative MTases from different clades show significant variation, indicating that these MTases might play roles in the survival of different bacteria (Fig. 4D). In addition, FlgJ is essential for bacterial flagellum formation. It has been previously demonstrated that the presence of FlgJ in phages can influence the survival rate of the bacterial host, as well as the growth and motility of bacterial cells (42, 43). These AMGs might be transferred to periodontal bacteria via phage-mediated processes, thereby influencing microbial metabolism, diversity, and evolution.

### Anti-prokaryotic immune systems are common and diverse in prophages

Phages and bacteria are engaged in a never-ending arms race (44). To successfully infect and incorporate their genomes into bacterial hosts, phages have evolved a set of anti-host defense mechanisms, also known as anti-prokaryotic defense systems. To investigate whether key periodontitis pathogens’ prophages encode anti-prokaryotic defense system genes, we performed a comprehensive analysis using dbAPIS, AntiDefenseFinder, and CRISPRdb from PhageScope.

**Fig 5:**
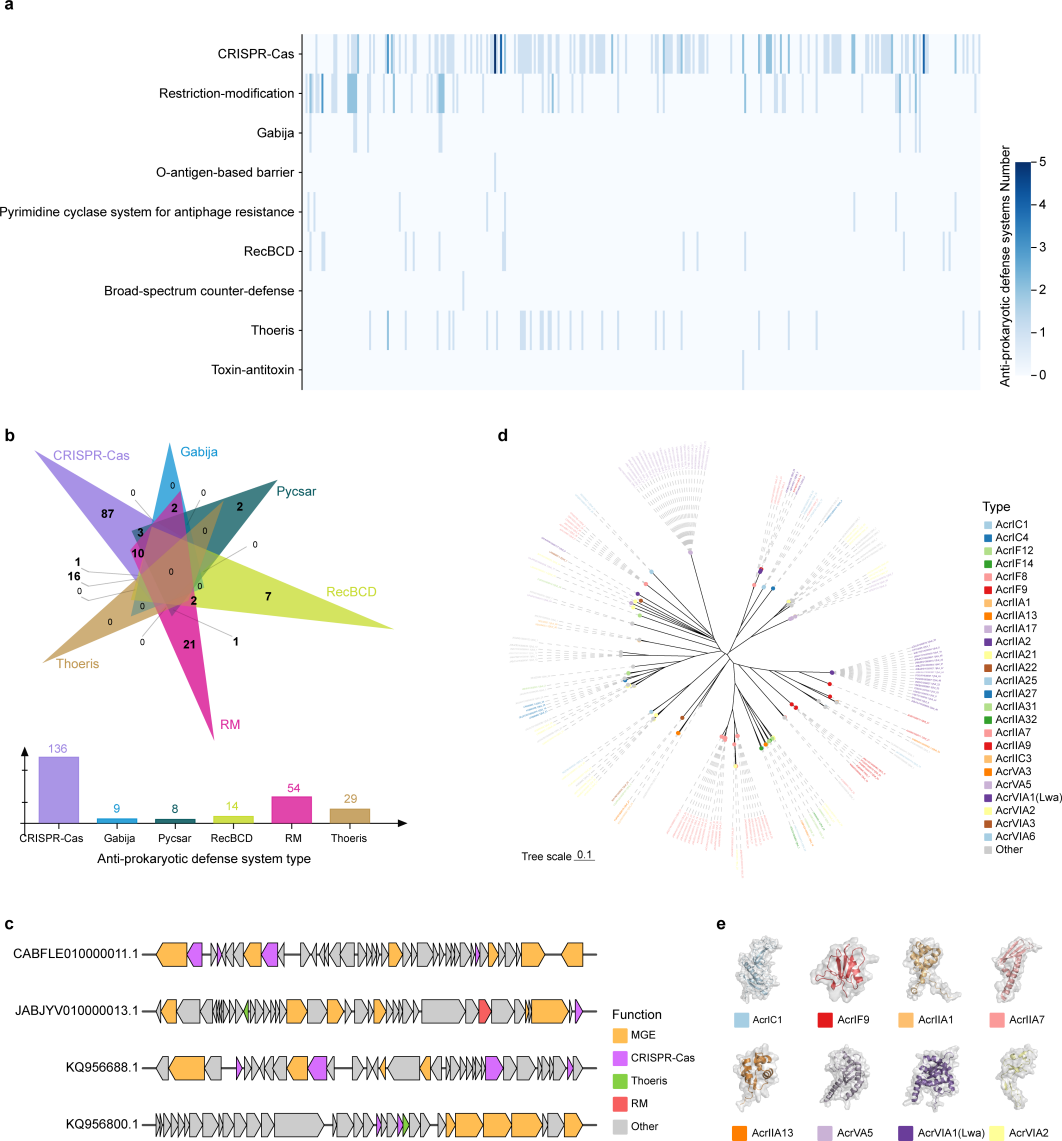
The anti-prokaryotic defense systems of prophages are diverse. (a) Heatmap analysis of the anti-prokaryotic defense system, corresponding to the reported defense systems. The frequencies of anti-prokaryotic defense systems are marked by different colors. (b) Venn diagram illustrating the number of unique and shared anti-prokaryotic defense system genes from prophages. (c) Local genome maps of prophages carrying the anti-prokaryotic defense system genes, with the genes for these anti-defense systems depicted in different colors, such as CRISPR-Cas (purple), Thoeris (green), RM (red). Meanwhile, other genes are also colored accordingly: MGEs (orange) and other phage genes (gray). (d) Phylogenetic tree of the anti-CRISPR genes, with the anti-CRISPR genes indicated in different colors. (e) Predicted structures of representative anti-CRISPR proteins in prophages.

As expected, genes encoding anti-prokaryotic immune systems were widely present in the prophages (44). In addition to providing resistance against common systems such as the RM, CRISPR-Cas, and TA, they also confer resistance against other defense mechanisms, including Gabija, O-antigen-based barrier, pyrimidine cyclase system for antiphage resistance (Pycsar), RecBCD, Thoeris, and broad-spectrum counter-defense (Fig. 5; Fig. S4a). The RM system (45) and the CRISPR-Cas system (46, 47) resist phage infection through mechanisms centered on recognizing, cutting, and degrading foreign DNA. The TA system (48, 49), Gabija (50), Pycsar (51), RecBCD (52), and Thoeris (53) are all associated with abortive infection defense mechanisms, mediating the death of host bacteria by activating various effector proteins within the bacteria to inhibit the replication of phages. The O-antigen-based barrier utilizes O antigens on the bacterial outer membrane surface to prevent the recognition of bacterial surfaces by phage receptor-binding proteins (54). Where there is defense, there is counter-defense. The key periodontitis pathogen prophage might make corresponding changes to a variety of different pathways to protect the phage from the attack of host bacteria and ensure its long-term integration in the host genome. For example, genes against the anti-Pycsar system can degrade cyclic nucleotides that activate defensive effectors, allowing phages to evade bacterial immune system attacks (51).

**Fig 6:**
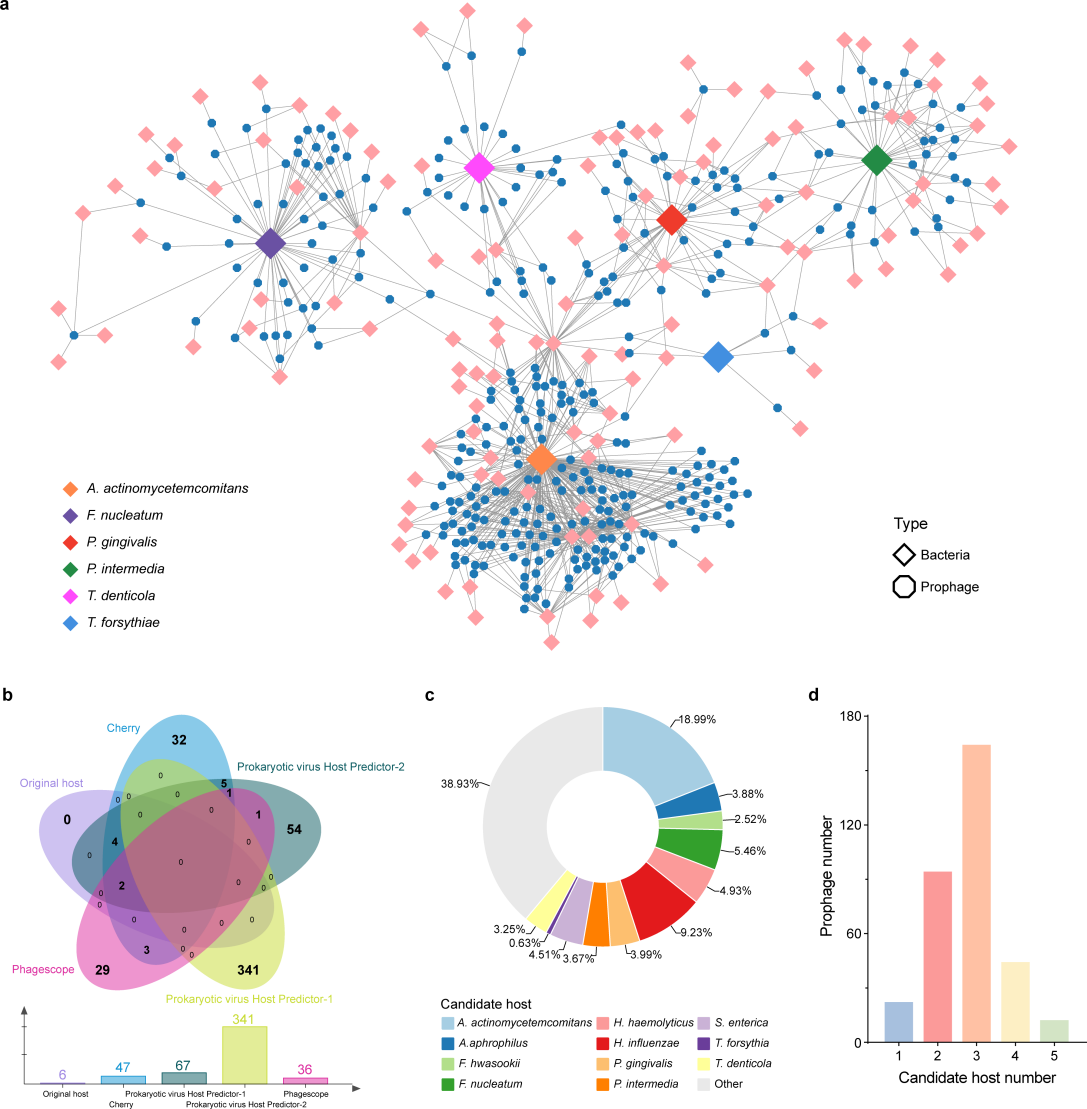
Potential hosts of prophages. (a) Phage-bacteria interaction network between prophage with candidate and actual host bacteria. In the network, phages are represented as blue octagonal blocks, and host bacteria are shown as diamond-shaped blocks in different colors. Edges between phage nodes and bacterial nodes represent potential infection relationships. (b) Venn diagram illustrating the number of unique and shared host bacteria of prophages. (c) Distribution of host bacteria in prophages. (d) Distribution of prophages with different numbers of candidate hosts.

The phage arms race has provided a wealth of candidate anti-CRISPRs (acrs), with over 100 acr genes experimentally verified (55). Across prophages, we identified 166 candidate acr genes (including 26 different types of anti-CRISPR). The phylogenetic tree grouped these 166 candidate acrs into different clusters (Fig. 5). Interestingly, five candidate acrs were found in the genome of phage LR698955.1-2, suggesting that host bacteria may try multiple CRISPR-Cas strategies to prevent phage infection (Fig. S4b). Representative structures of acrs demonstrated the functional diversity of anti-CRISPR systems (Fig. 5). The identification of these anti-nucleic immune systems in the prophages of periodontitis pathogens reveals how phages persist in a competitive microbial environment and further provides a rich pool of candidate genes for characterizing the phage-bacteria arms race in the oral microbiome.

### Cross-infection of phages and bacteria constitutes the interaction network

In the human oral microbiome, the number of phages far exceeds bacteria (14). It is possible for a host to be frequently infected by more than one phage, resulting in changes in gene exchange between phages and affecting the host spectrum of phages (56). To further investigate the interactions between prophages and their host bacteria, we employed various advanced tools to infer the candidate host bacteria of these prophages. Surprisingly, these phages have the potential to infect a variety of different host bacteria (Fig. 6a through c). Based on the predicted hosts identified and the actual host in this study, we constructed a phage-bacteria interaction network, where the nodes and edges represent the phages/bacteria and their invasion relationships, respectively, forming an interaction network (Fig. 6a). In this network, 133 different predicted bacterial species and actual bacterial hosts (represented as diamond-shaped blocks) were connected by phages (represented as octagonal blocks). These phages and bacteria formed a large intersecting cluster, with the most densely connected region centered around *A. actinomycetemcomitans*. Remarkably, we observed that these phages were not only linked to actual bacterial hosts; most phages were connected to one or more predicted bacterial species (Fig. 6a and d). For example, phage JAGZII010000159.1 was linked to four predicted bacterial species: *P. harei*, R. stabekisii, *L. boronitolerans*, and *V. parvula*. Interestingly, we also observed that phage VSDT01000005.1 might simultaneously infect both the pathogenic bacterium *A. actinomycetemcomitans* and the commensal bacteria *P. excrementihominis* and *S. oralis*. This suggests that phages may play a crucial role in regulating both pathogenic and commensal bacterial communities, contributing to the formation of periodontal microbial populations and communities.

## DISCUSSION

The human oral microbiome plays a critical role in maintaining oral health and driving disease progression (3). Viruses are regarded as key regulators of diverse ecological niches in many ecosystems (4). In the periodontal ecosystem, phages dominate the microbiome, with about 10^10^ phages per milligram of dental plaque (57). Surprisingly, our understanding of phages within the periodontal microecosystem remains limited (9, 16). Here, we systematically characterize the prophages present in the genomes of the key periodontitis pathogens. Using a variety of advanced bioinformatics tools, we annotated the ORFs encoded by the prophages. We further explored the functional gene repertoire related to the arms race between prophages and their hosts. We found that these prophages not only encode genes related to their survival but also encode various host-associated auxiliary function genes and mobile elements.

The oral microbiome is considered a reservoir of antibiotic resistance (30). In this study, we identified a large number of ARGs within the prophages, involving a variety of different antibiotics. The dense structure of microbial communities in periodontal biofilms provides an ideal environment for the dissemination of ARGs. Oral resistance to tetracycline, amoxicillin, and metronidazole has been identified in oral plaque samples (58). In addition, we discovered various VFs in the prophages, which may play an important role in the physiology of the host bacteria. Integrated prophages have been shown to add genes that enhance host survival or more effective pathogenicity to the virulence factor repertoire of bacterial hosts (59). For instance, *Vibrio cholerae*, *Corynebacterium diphtheriae*, and *Shiga toxin-producing Escherichia coli* rely on specific prophage-encoded VFs to cause certain diseases, while *Staphylococcus aureus* and *Salmonella Typhimurium* contain numerous prophages, each of which encodes virulence or fitness factors that incrementally contribute to the fitness of the lysogen. Thus, the transformation of phage lysogeny could alter the overall ecology and virulence of the microbial community by converting otherwise harmless bacteria into a pathogenic state through the provision of virulence factors. In addition, we identified abundant CAZymes within these prophages. Recent metagenomic studies of the pig gut microbiome have shown that bacteria obtaining CAZymes from gut phage reservoirs might gain enhanced foraging capabilities under nutrient-poor conditions (60). This suggests that periodontal pathogens could potentially utilize phage-derived genes to utilize carbon sources from various origins to promote their survival and growth.

Horizontal gene transfer has already been established as a crucial mechanism for gene dissemination and acquisition among different bacterial populations in the oral cavity (61). The identification of MGEs within the prophages further reinforces the idea that phages are important players in regulating the virulence of key periodontitis pathogens and their interactions with host cells. The various auxiliary genes identified in the prophages in this study indicate that phages might be essential in driving the dysbiosis of the periodontal microbiome.

The co-evolution of phages and their bacterial hosts has driven the emergence and diversification of defense and counter-defense systems. Identifying anti-prokaryotic immune systems within phages can offer deeper insights into the host-pathogen arms race. Most bacterial genomes encode more than two defense systems, while phages have also evolved both direct and indirect immune systems to bypass or block bacterial defense pathways and ensure successful infection (62, 63). In the key periodontitis pathogen prophages, multiple anti-prokaryotic immune systems were widely detected. Among them, anti-restriction-modification and acr proteins were the most prevalent, which can protect against nucleic acid targeting systems. In addition, we observed that phages also contain various other types of anti-prokaryotic immune systems, including not only abortive infection systems but also those involving nucleic acid modifications and protein modifications or degradation. These diverse anti-prokaryotic immune strategies may work together to protect phages from the diverse attacks of host bacteria to achieve phage immune evasion. Anti-prokaryotic immune systems have been identified across diverse ecological environments. For instance, Dong et al. discovered a substantial and diverse viral population in deep-sea cold seep sediments, which had evolved adaptive strategies to counter host bacterial defenses, including acr proteins and antitoxins (64). The interplay between defense and counter-defense systems reveals the intricate antagonistic interactions between phages and host bacteria in the periodontal environment, suggesting that phages may play a role in both intra- and interspecies antagonism within the periodontal microbiome.

Next, this study predicts the host range of these prophages, which is significant for assessing the potential drivers of phage influence on the clinical progression or mitigation of periodontal disease. Surprisingly, only a few phages are linked to a single bacterial host; the majority are associated with multiple bacterial hosts. This indicates a possibility of cross-infection among the key periodontitis pathogen phages. Through metagenomic analysis, Zhao et al. discovered that phages present in the oral ecosystem can cross-infect both periodontal pathogens and commensal bacteria, suggesting that these phages may regulate the symbiotic microbiome while directly or indirectly inhibiting the growth of pathogenic bacteria (11). Moreover, cross-infecting phages have been found in studies of other ecosystems. For example, cyanophages that infect the *cyanobacterium Prochlorococcus* can also target *Synechococcus* (65). In the phage-bacteria interaction network of this study, we also observed that some phages might simultaneously infect *P. gingivalis* and *P. intermedia*, potentially facilitating the exchange of virulence genes among pathogens. The cross-infection phenomenon of phages and the annotation results of various auxiliary genes suggest that phages may regulate the survival and metabolism of their hosts at multiple levels by transferring virulence genes, antibiotic resistance genes, and other auxiliary metabolic genes. This interaction can shape the periodontal microbiome and pose potential risks to the periodontal microbiome.

In this study, there are several issues that warrant further exploration. The periodontal microbiota consists of a large number of bacteria, including a substantial number of uncharacterized phages (25, 26, 66). However, the relatively small sample size and limited scope of this study may impact the generalizability and representativeness of the results. To gain a more comprehensive understanding of the role of phages in periodontal disease, future research should simultaneously analyze and compare phages within both pathogenic and commensal bacteria, thereby revealing the potential impact of prophages from different species on periodontal health (16, 26). In addition, this study lacks an analysis of the correlation between specific bacteria and the clinical conditions, preventing direct linkage of the results to real disease states. These limitations may hinder our understanding of the precise role of phages in periodontal disease. At present, our study primarily relies on predicted prophage sequences, but the accuracy of phage predictions is constrained by the limitations of the algorithms used. VirSorter2, which utilizes machine learning models and database comparisons to extract features from sequences and classify them, helps identify viral sequences (67). However, if key genes of a prophage or prophage-like genes have not yet been included in existing databases, the prophage may be misclassified as incomplete or even undetected (68–70). This could lead to an underestimation of the number of phages present.

Phages play a pivotal role in influencing the adaptability and virulence of bacterial hosts (5, 11), potentially altering bacterial evolutionary trajectories through gene transfer across species (4, 5). Given the diversity of phages and their potential host ranges, understanding these interactions is of paramount importance. However, a significant challenge remains in accurately predicting prophage-host relationships, as current tools still exhibit notable limitations (69, 71–74). Despite the progress made in predictive algorithms, discrepancies in accuracy across different methods highlight the inherent uncertainty in host prediction. Specifically, CHERRY achieved an accuracy of 78% (72) in predicting host species, while PhageScope reached 83% (74) in validation accuracy. By contrast, the Prokaryotic Virus Host Predictors (1 and 2) exhibited significantly lower accuracy, with predictions at the genus level being as low as 29% and 33%, respectively, and only slightly better at the family level, with accuracy reaching 64% and 75% (73). These discrepancies underscore the challenge of achieving reliable prophage-host predictions, as different training data sets and prediction methodologies can lead to divergent results. In the context of this study, the host predictions may, therefore, be viewed as transitional or provisional. While these findings are valuable, they should be interpreted with caution, as the current tools do not yet offer fully reliable predictions at all taxonomic levels. Nonetheless, it is important to recognize that advancements in research methodologies, data set expansions, and algorithm refinements are likely to improve prediction accuracy in the future. As such, the results of this study provide a stepping stone for future work, and we anticipate that continued progress in this field will enable more precise and dependable analyses of phage-host interactions.

In summary, the findings regarding the key periodontitis pathogens prophages confirm that phages play a crucial role in shaping the periodontal ecosystem. Future research will need more sampling and analysis to elucidate the specific roles of phages in periodontal health and disease. The genomic analysis of key periodontitis pathogens prophages provides essential supplementary data for studying periodontal phages, revealing new insights into their fundamental role in the periodontal ecosystem.

## MATERIALS AND METHODS

### Detection of prophages in key periodontitis pathogens

Complete genomes of key periodontal pathogens, including *Porphyromonas gingivalis*, *Fusobacterium nucleatum*, *Treponema denticola*, *Tannerella forsythia*, *Prevotella intermedia*, and *Aggregatibacter actinomycetemcomitans*, were retrieved from the National Center for Biotechnology Information (NCBI) database (https://www.ncbi.nlm.nih.gov/) (last accessed in January 2022). To validate the prophage predictions, we followed a publicly available protocol (dx.doi.org/10.17504/protocols.io.bwm5pc86). Briefly, VirSorter2 was used to perform a secondary screening of prophages processed by CheckV to ensure reliability and accuracy. Specifically, VirSorter2 (67) was employed to analyze the whole-genome sequences of all downloaded strains for prophage identification (minimum score: 0.5, length ≥5 kb) (https://github.com/jiarong/VirSorter2). The identified prophages were further processed using CheckV (75) (https://bitbucket.org/berkeleylab/CheckV/src). Sequences that did not meet the quality standards were removed based on default parameters, while the remaining prophages were resubmitted to VirSorter2 using the same parameters as in the initial analysis. Strain details are provided in Table S1A, and prophage information is listed in Table S1B.

### Prophage genome analysis

Identified prophage genomes were further analyzed using various tools. Briefly, a phylogenetic tree was constructed using PhageScope (74) (https://phagescope.deepomics.org/), and intergenomic similarity analysis was generated with VIRIDIC (https://rhea.icbm.uni-oldenburg.de/viridic/). The phylogenetic tree was visualized using tvBOT (76) (https://www.chiplot.online/). BLAST was used to compare the prophage genomes against the viral database from the IMG/VR v4 database (28) with default parameters. Taxonomic classification, ORFs, and structural annotation were performed in PhageScope using HMMsearch, Prodigal v2.6.3, and Eggnog-mapper v2.1.10, respectively. Standard PhageScope parameters were used for all analyses.

### Identification of antibiotic resistance genes, virulence factors, mobile genetic elements, and carbohydrate-active enzymes carried by prophages

Identification of antibiotic resistance genes, virulence factors, mobile genetic elements, and carbohydrate-active enzymes carried by prophages was conducted using various specialized databases. Virulence factors (VF) were identified through the Virulence Factors of Pathogenic Bacteria Database (VFDB) (77) (http://www.mgc.ac.cn/VFs/), while antimicrobial resistance (AMR) genes were analyzed using the Comprehensive Antibiotic Resistance Database (CARD) (78) (https://card.mcmaster.ca/). MGEs were identified via the mobile orthologous groups database (mobileOG-db) (79) (https://mobileogdb.flsi.cloud.vt.edu/), and carbohydrate-active enzymes (CAZymes) were annotated using the dbCAN3 (80) database (https://bcb.unl.edu/dbCAN2/). Visualizations were created as follows: gene clusters and pie charts were illustrated with tvBOT, Venn diagrams with EVenn (81) (http://www.ehbio.com/test/venn/#/), and CAZyme heatmaps also in tvBOT.

### Identification of auxiliary metabolic genes

AMGs were predicted on the prophage genomes using the DRAM-v module from DRAM (82) with default parameters. To avoid reporting potential false positives, AMGs were only considered for metabolic genes (M) and metabolic genes situated between at least two phage genes (DRAM-v AMG score 1, 2). BLASTP was used to query the sequence of DNA (cytosine-5)-methyltransferase (MTases) against the NCBI RefSeq database with default settings, to obtain the top 10 hits as the reference sequences. Schematic diagrams of the AMG gene cluster and pie plot were visualized in tvBOT. The phylogenetic tree of the viral and microbial MTases genes was generated using Clustal Omega (83) (https://www.ebi.ac.uk/jdispatcher/msa/clustalo), and then visualized in tvBOT.

### Identification of anti-prokaryotic defense genes

Anti-prokaryotic defense genes were identified within prophage genomes using multiple databases: the database of anti-prokaryotic immune system (dbAPIS) (62) (https://bcb.unl.edu/dbAPIS/index.php), CRISPRdb in PhageScore, and AntiDefenseFinder (63) (https://defensefinder.mdmlab.fr/), with all analyses conducted using standard parameters. A phylogenetic tree of anti-CRISPR (acr) genes was constructed using Clustal Omega. Visualizations were prepared as follows: the heatmap, gene clusters, and phylogenetic tree for anti-prokaryotic defense systems were generated in tvBOT, and Venn diagrams were created using EVenn.

### Protein 3D structure prediction

The 3D structures of MTases and acr proteins were predicted using AlphaFold2 (v2.4.5) (84) with default parameters. Confidence levels and per-residue metrics were evaluated to confirm model reliability. Structures were subsequently visualized and analyzed in PyMOL (v3.0.5) (85), where secondary structure features and binding sites were annotated as needed.

### Prediction of prophage hosts

The hosts of prophages were predicted by CHERRY in PhaBOX (86) (https://phage.ee.cityu.edu.hk/), DeepHost in PhageScore (74), and Prokaryotic virus Host Predictor (PHP) (73) (Prokaryotic virus Host Predictor-1, host with the largest score; Prokaryotic virus Host Predictor-2, host with the top five consensus) (http://www.computationalbiology.cn/phageHostPredictor/home.html). Standard parameters were used for all analyses. A manually curated phage-host data set was visualized in Cytoscape v3.10.2 (87), while Venn diagrams for host predictions from different tools were generated using EVenn. A pie chart showing candidate hosts for prophages was created in tvBOT.

### Statistical analysis

Pearson’s correlation tests were performed in SPSS v26.0.0. Statistical significance was set at *P*  <  0.05.

## SUPPLEMENTAL MATERIAL

Fig. S1 msystems.00201-25-s0001.tif Whole-genome maps of representative prophages containing VF, ARG, MGE, and prophage hallmark genes.

Fig. S2 msystems.00201-25-s0002.tif Characterization of auxiliary metabolic genes in prophages.

Fig. S3 msystems.00201-25-s0003.tif Whole-genome maps of representative prophages carrying the auxiliary metabolic genes.

Fig. S4 msystems.00201-25-s0004.tif Whole-genome maps of prophages carrying the anti-prokaryotic defense system genes.

Legends msystems.00201-25-s0005.docx Legends for Fig. S1 to S4.

Table S1 msystems.00201-25-s0006.xlsx The data of each bacterial genome and prophage.

ASM does not own the copyrights to Supplemental Material that may be linked to, or accessed through, an article. The authors have granted ASM a non-exclusive, world-wide license to publish the Supplemental Material files. Please contact the corresponding author directly for reuse.

## References

[1] Slots J. 2017. Periodontitis: facts, fallacies and the future. Periodontol 2000 75:7–23. doi:10.1111/prd.12221 28758294

[2] Di Stefano M, Polizzi A, Santonocito S, Romano A, Lombardi T, Isola G. 2022. Impact of oral microbiome in periodontal health and periodontitis: a critical review on prevention and treatment. Int J Mol Sci 23:5142. doi:10.3390/ijms23095142 35563531 PMC9103139

[3] Baker JL, Mark Welch JL, Kauffman KM, McLean JS, He X. 2024. The oral microbiome: diversity, biogeography and human health. Nat Rev Microbiol 22:89–104. doi:10.1038/s41579-023-00963-6 37700024 PMC11084736

[4] Chevallereau A, Pons BJ, van Houte S, Westra ER. 2022. Interactions between bacterial and phage communities in natural environments. Nat Rev Microbiol 20:49–62. doi:10.1038/s41579-021-00602-y 34373631

[5] Fortier LC, Sekulovic O. 2013. Importance of prophages to evolution and virulence of bacterial pathogens. Virulence 4:354–365. doi:10.4161/viru.24498 23611873 PMC3714127

[6] Hu J, Ye H, Wang S, Wang J, Han D. 2021. Prophage activation in the intestine: insights into functions and possible applications. Front Microbiol 12:785634. doi:10.3389/fmicb.2021.785634 34966370 PMC8710666

[7] Mahmud MR, Tamanna SK, Akter S, Mazumder L, Akter S, Hasan MR, Acharjee M, Esti IZ, Islam MS, Shihab MMR, Nahian M, Gulshan R, Naser S, Pirttilä AM. 2024. Role of bacteriophages in shaping gut microbial community. Gut Microbes 16:2390720. doi:10.1080/19490976.2024.2390720 39167701 PMC11340752

[8] Wendling CC, Refardt D, Hall AR. 2021. Fitness benefits to bacteria of carrying prophages and prophage-encoded antibiotic-resistance genes peak in different environments. Evolution 75:515–528. doi:10.1111/evo.14153 33347602 PMC7986917

[9] Shanmugasundaram S, Nayak N, Puzhankara L, Kedlaya MN, Rajagopal A, Karmakar S. 2024. Bacteriophages: the dawn of a new era in periodontal microbiology? Crit Rev Microbiol 50:212–223. doi:10.1080/1040841X.2023.2182667 36883683

[10] Ly M, Abeles SR, Boehm TK, Robles-Sikisaka R, Naidu M, Santiago-Rodriguez T, Pride DT. 2014. Altered oral viral ecology in association with periodontal disease. mBio 5:e01133-14. doi:10.1128/mBio.01133-14 24846382 PMC4030452

[11] Wang J, Gao Y, Zhao F. 2016. Phage-bacteria interaction network in human oral microbiome. Environ Microbiol 18:2143–2158. doi:10.1111/1462-2920.12923 26036920

[12] Jahn MT, Arkhipova K, Markert SM, Stigloher C, Lachnit T, Pita L, Kupczok A, Ribes M, Stengel ST, Rosenstiel P, Dutilh BE, Hentschel U. 2019. A phage protein aids bacterial symbionts in eukaryote immune evasion. Cell Host Microbe 26:542–550. doi:10.1016/j.chom.2019.08.019 31561965

[13] Champagne-Jorgensen K, Luong T, Darby T, Roach DR. 2023. Immunogenicity of bacteriophages. Trends Microbiol 31:1058–1071. doi:10.1016/j.tim.2023.04.008 37198061

[14] Martínez A, Kuraji R, Kapila YL. 2021. The human oral virome: shedding light on the dark matter. Periodontol 2000 87:282–298. doi:10.1111/prd.12396 34463988 PMC8457075

[15] Yahara K, Suzuki M, Hirabayashi A, Suda W, Hattori M, Suzuki Y, Okazaki Y. 2021. Long-read metagenomics using PromethION uncovers oral bacteriophages and their interaction with host bacteria. Nat Commun 12:27. doi:10.1038/s41467-020-20199-9 33397904 PMC7782811

[16] Łasica A, Golec P, Laskus A, Zalewska M, Gędaj M, Popowska M. 2024. Periodontitis: etiology, conventional treatments, and emerging bacteriophage and predatory bacteria therapies. Front Microbiol 15:1469414. doi:10.3389/fmicb.2024.1469414 39391608 PMC11464445

[17] Matrishin CB, Haase EM, Dewhirst FE, Mark Welch JL, Miranda-Sanchez F, Chen T, MacFarland DC, Kauffman KM. 2023. Phages are unrecognized players in the ecology of the oral pathogen Porphyromonas gingivalis. Microbiome 11:161. doi:10.1186/s40168-023-01607-w 37491415 PMC10367356

[18] Gu BL, She Y, Pei GK, Du Y, Yang R, Ma LX, Zhao Q, Gao SG. 2023. Systematic analysis of prophages carried by Porphyromonas gingivalis. Infect Genet Evol 113:105489. doi:10.1016/j.meegid.2023.105489 37572952

[19] Łobocka M, Dąbrowska K, Górski A. 2021. Engineered bacteriophage therapeutics: rationale, challenges and future. BioDrugs 35:255–280. doi:10.1007/s40259-021-00480-z 33881767 PMC8084836

[20] Moraru C, Varsani A, Kropinski AM. 2020. VIRIDIC-A novel tool to calculate the intergenomic similarities of prokaryote-infecting viruses. Viruses 12:1268. doi:10.3390/v12111268 33172115 PMC7694805

[21] Chibani-Chennoufi S, Bruttin A, Dillmann ML, Brüssow H. 2004. Phage-host interaction: an ecological perspective. J Bacteriol 186:3677–3686. doi:10.1128/JB.186.12.3677-3686.2004 15175280 PMC419959

[22] Koskella B, Brockhurst MA. 2014. Bacteria-phage coevolution as a driver of ecological and evolutionary processes in microbial communities. FEMS Microbiol Rev 38:916–931. doi:10.1111/1574-6976.12072 24617569 PMC4257071

[23] Mavrich TN, Hatfull GF. 2017. Bacteriophage evolution differs by host, lifestyle and genome. Nat Microbiol 2:17112. doi:10.1038/nmicrobiol.2017.112 28692019 PMC5540316

[24] Hatfull GF, Hendrix RW. 2011. Bacteriophages and their genomes. Curr Opin Virol 1:298–303. doi:10.1016/j.coviro.2011.06.009 22034588 PMC3199584

[25] Curtis MA, Diaz PI, Van Dyke TE. 2020. The role of the microbiota in periodontal disease. Periodontol 2000 83:14–25. doi:10.1111/prd.12296 32385883

[26] Guo X, Wang X, Shi J, Ren J, Zeng J, Li J, Li Y. 2024. A review and new perspective on oral bacteriophages: manifestations in the ecology of oral diseases. J Oral Microbiol 16:2344272. doi:10.1080/20002297.2024.2344272 38698893 PMC11064738

[27] Li S, Guo R, Zhang Y, Li P, Chen F, Wang X, Li J, Jie Z, Lv Q, Jin H, Wang G, Yan Q. 2022. A catalog of 48,425 nonredundant viruses from oral metagenomes expands the horizon of the human oral virome. iScience 25:104418. doi:10.1016/j.isci.2022.104418 35663034 PMC9160773

[28] Camargo AP, Nayfach S, Chen I-MA, Palaniappan K, Ratner A, Chu K, Ritter SJ, Reddy TBK, Mukherjee S, Schulz F, Call L, Neches RY, Woyke T, Ivanova NN, Eloe-Fadrosh EA, Kyrpides NC, Roux S. 2023. IMG/VR v4: an expanded database of uncultivated virus genomes within a framework of extensive functional, taxonomic, and ecological metadata. Nucleic Acids Res 51:D733–D743. doi:10.1093/nar/gkac1037 36399502 PMC9825611

[29] Olsvik B, Tenover FC. 1993. Tetracycline resistance in periodontal pathogens. Clin Infect Dis 16:S310–S313. doi:10.1093/clinids/16.supplement_4.s310 8324137

[30] Brooks L, Narvekar U, McDonald A, Mullany P. 2022. Prevalence of antibiotic resistance genes in the oral cavity and mobile genetic elements that disseminate antimicrobial resistance: a systematic review. Mol Oral Microbiol 37:133–153. doi:10.1111/omi.12375 35674142

[31] Ng E, Tay JRH, Boey SK, Laine ML, Ivanovski S, Seneviratne CJ. 2024. Antibiotic resistance in the microbiota of periodontitis patients: an update of current findings. Crit Rev Microbiol 50:329–340. doi:10.1080/1040841X.2023.2197481 37140235

[32] Teughels W, Feres M, Oud V, Martín C, Matesanz P, Herrera D. 2020. Adjunctive effect of systemic antimicrobials in periodontitis therapy: a systematic review and meta-analysis. J Clin Periodontol 47:257–281. doi:10.1111/jcpe.13264 31994207

[33] Harvey KL, Jarocki VM, Charles IG, Djordjevic SP. 2019. The diverse functional roles of elongation factor Tu (EF-Tu) in microbial pathogenesis. Front Microbiol 10:2351. doi:10.3389/fmicb.2019.02351 31708880 PMC6822514

[34] Torres AN, Chamorro-Veloso N, Costa P, Cádiz L, Del Canto F, Venegas SA, López Nitsche M, Coloma-Rivero RF, Montero DA, Vidal RM. 2020. Deciphering additional roles for the EF-Tu, l-asparaginase II and OmpT proteins of shiga toxin-producing Escherichia coli. Microorganisms 8:1184. doi:10.3390/microorganisms8081184 32759661 PMC7464798

[35] Xiong C, Jiao H, Ran J, Li D, Li Z, Wang B, Luo H, Li Y, Lin Y, Yao J, Wu R. 2025. A comprehensive understanding of the influence and molecular mechanism of exeA on the pathogenicity in Aeromonas hydrophila. Int J Biol Macromol 284:138080. doi:10.1016/j.ijbiomac.2024.138080 39603288

[36] Howard SP, Meiklejohn HG, Shivak D, Jahagirdar R. 1996. A TonB-like protein and A novel membrane protein containing an ATP-binding cassette function together in exotoxin secretion. Mol Microbiol 22:595–604. doi:10.1046/j.1365-2958.1996.d01-1713.x 8951808

[37] Tian F, Wainaina JM, Howard-Varona C, Domínguez-Huerta G, Bolduc B, Gazitúa MC, Smith G, Gittrich MR, Zablocki O, Cronin DR, Eveillard D, Hallam SJ, Sullivan MB. 2024. Prokaryotic-virus-encoded auxiliary metabolic genes throughout the global oceans. Microbiome 12:159. doi:10.1186/s40168-024-01876-z 39198891 PMC11360552

[38] Luo X-Q, Wang P, Li J-L, Ahmad M, Duan L, Yin L-Z, Deng Q-Q, Fang B-Z, Li S-H, Li W-J. 2022. Viral community-wide auxiliary metabolic genes differ by lifestyles, habitats, and hosts. Microbiome 10:190. doi:10.1186/s40168-022-01384-y 36333738 PMC9636769

[39] Murphy J, Mahony J, Ainsworth S, Nauta A, van Sinderen D. 2013. Bacteriophage orphan DNA methyltransferases: insights from their bacterial origin, function, and occurrence. Appl Environ Microbiol 79:7547–7555. doi:10.1128/AEM.02229-13 24123737 PMC3837797

[40] Sun C, Chen J, Jin M, Zhao X, Li Y, Dong Y, Gao N, Liu Z, Bork P, Zhao X-M, Chen W-H. 2023. Long-read sequencing reveals extensive DNA methylations in human gut phagenome contributed by prevalently phage-encoded methyltransferases. Adv Sci (Weinh) 10:e2302159. doi:10.1002/advs.202302159 37382405 PMC10477858

[41] Gao Q, Lu S, Wang Y, He L, Wang M, Jia R, Chen S, Zhu D, Liu M, Zhao X, Yang Q, Wu Y, Zhang S, Huang J, Mao S, Ou X, Sun D, Tian B, Cheng A. 2023. Bacterial DNA methyltransferase: a key to the epigenetic world with lessons learned from proteobacteria. Front Microbiol 14:1129437. doi:10.3389/fmicb.2023.1129437 37032876 PMC10073500

[42] Coloma-Rivero RF, Gómez L, Alvarez F, Saitz W, Del Canto F, Céspedes S, Vidal R, Oñate AA. 2020. The role of the flagellar protein FlgJ in the virulence of Brucella abortus. Front Cell Infect Microbiol 10:178. doi:10.3389/fcimb.2020.00178 32411617 PMC7198779

[43] Luo S, Ru J, Mirzaei MK, Xue J, Peng X, Ralser A, Mejías-Luque R, Gerhard M, Deng L. 2023. Gut virome profiling identifies an association between temperate phages and colorectal cancer promoted by Helicobacter pylori infection. Gut Microbes 15:2257291. doi:10.1080/19490976.2023.2257291 37747149 PMC10578192

[44] Hampton HG, Watson BNJ, Fineran PC. 2020. The arms race between bacteria and their phage foes. Nature 577:327–336. doi:10.1038/s41586-019-1894-8 31942051

[45] Birkholz N, Jackson SA, Fagerlund RD, Fineran PC. 2022. A mobile restriction–modification system provides phage defence and resolves an epigenetic conflict with an antagonistic endonuclease. Nucleic Acids Res 50:3348–3361. doi:10.1093/nar/gkac147 35286398 PMC8989522

[46] Pawluk A, Davidson AR, Maxwell KL. 2018. Anti-CRISPR: discovery, mechanism and function. Nat Rev Microbiol 16:12–17. doi:10.1038/nrmicro.2017.120 29062071

[47] Georjon H, Bernheim A. 2023. The highly diverse antiphage defence systems of bacteria. Nat Rev Microbiol 21:686–700. doi:10.1038/s41579-023-00934-x 37460672

[48] LeRoux M, Laub MT. 2022. Toxin-antitoxin systems as phage defense elements. Annu Rev Microbiol 76:21–43. doi:10.1146/annurev-micro-020722-013730 35395167

[49] Kelly A, Arrowsmith TJ, Went SC, Blower TR. 2023. Toxin-antitoxin systems as mediators of phage defence and the implications for abortive infection. Curr Opin Microbiol 73:102293. doi:10.1016/j.mib.2023.102293 36958122

[50] Antine SP, Johnson AG, Mooney SE, Leavitt A, Mayer ML, Yirmiya E, Amitai G, Sorek R, Kranzusch PJ. 2024. Structural basis of Gabija anti-phage defence and viral immune evasion. Nature 625:360–365. doi:10.1038/s41586-023-06855-2 37992757 PMC10781630

[51] Hobbs SJ, Wein T, Lu A, Morehouse BR, Schnabel J, Leavitt A, Yirmiya E, Sorek R, Kranzusch PJ. 2022. Phage anti-CBASS and anti-Pycsar nucleases subvert bacterial immunity. Nature 605:522–526. doi:10.1038/s41586-022-04716-y 35395152 PMC9117128

[52] Millman A, Bernheim A, Stokar-Avihail A, Fedorenko T, Voichek M, Leavitt A, Oppenheimer-Shaanan Y, Sorek R. 2020. Bacterial retrons function in anti-phage defense. Cell 183:1551–1561. doi:10.1016/j.cell.2020.09.065 33157039

[53] Hobbs SJ, Kranzusch PJ. 2024. Nucleotide immune signaling in CBASS, Pycsar, Thoeris, and CRISPR antiphage defense. Annu Rev Microbiol 78:255–276. doi:10.1146/annurev-micro-041222-024843 39083849 PMC12335278

[54] Letarov AV. 2023. Bacterial virus forcing of bacterial O-antigen shields: lessons from coliphages. Int J Mol Sci 24:17390. doi:10.3390/ijms242417390 38139217 PMC10743462

[55] Bondy-Denomy J, Davidson AR, Doudna JA, Fineran PC, Maxwell KL, Moineau S, Peng X, Sontheimer EJ, Wiedenheft B. 2018. A unified resource for tracking anti-CRISPR names. CRISPR J 1:304–305. doi:10.1089/crispr.2018.0043 31021273 PMC10625466

[56] de Jonge PA, Nobrega FL, Brouns SJJ, Dutilh BE. 2019. Molecular and evolutionary determinants of bacteriophage host range. Trends Microbiol 27:51–63. doi:10.1016/j.tim.2018.08.006 30181062

[57] Edlund A, Santiago-Rodriguez TM, Boehm TK, Pride DT. 2015. Bacteriophage and their potential roles in the human oral cavity. J Oral Microbiol 7:27423. doi:10.3402/jom.v7.27423 25861745 PMC4393417

[58] Carr VR, Witherden EA, Lee S, Shoaie S, Mullany P, Proctor GB, Gomez-Cabrero D, Moyes DL. 2020. Abundance and diversity of resistomes differ between healthy human oral cavities and gut. Nat Commun 11:693. doi:10.1038/s41467-020-14422-w 32019923 PMC7000725

[59] Brüssow H, Canchaya C, Hardt WD. 2004. Phages and the evolution of bacterial pathogens: from genomic rearrangements to lysogenic conversion. Microbiol Mol Biol Rev 68:560–602. doi:10.1128/MMBR.68.3.560-602.2004 15353570 PMC515249

[60] Hu J, Chen J, Nie Y, Zhou C, Hou Q, Yan X. 2024. Characterizing the gut phageome and phage-borne antimicrobial resistance genes in pigs. Microbiome 12:102. doi:10.1186/s40168-024-01818-9 38840247 PMC11151549

[61] Roberts AP, Kreth J. 2014. The impact of horizontal gene transfer on the adaptive ability of the human oral microbiome. Front Cell Infect Microbiol 4:124. doi:10.3389/fcimb.2014.00124 25250243 PMC4157583

[62] Yan Y, Zheng J, Zhang X, Yin Y. 2024. dbAPIS: a database of anti-prokaryotic immune system genes. Nucleic Acids Res 52:D419–D425. doi:10.1093/nar/gkad932 37889074 PMC10767833

[63] Tesson F, Hervé A, Mordret E, Touchon M, d’Humières C, Cury J, Bernheim A. 2022. Systematic and quantitative view of the antiviral arsenal of prokaryotes. Nat Commun 13:2561. doi:10.1038/s41467-022-30269-9 35538097 PMC9090908

[64] Peng Y, Lu Z, Pan D, Shi L-D, Zhao Z, Liu Q, Zhang C, Jia K, Li J, Hubert CRJ, Dong X. 2023. Viruses in deep-sea cold seep sediments harbor diverse survival mechanisms and remain genetically conserved within species. ISME J 17:1774–1784. doi:10.1038/s41396-023-01491-0 37573455 PMC10504277

[65] Sullivan MB, Waterbury JB, Chisholm SW. 2003. Cyanophages infecting the oceanic cyanobacterium Prochlorococcus. Nature 424:1047–1051. doi:10.1038/nature01929 12944965

[66] Peng X, Cheng L, You Y, Tang C, Ren B, Li Y, Xu X, Zhou X. 2022. Oral microbiota in human systematic diseases. Int J Oral Sci 14:14. doi:10.1038/s41368-022-00163-7 35236828 PMC8891310

[67] Guo J, Bolduc B, Zayed AA, Varsani A, Dominguez-Huerta G, Delmont TO, Pratama AA, Gazitúa MC, Vik D, Sullivan MB, Roux S. 2021. VirSorter2: a multi-classifier, expert-guided approach to detect diverse DNA and RNA viruses. Microbiome 9:37. doi:10.1186/s40168-020-00990-y 33522966 PMC7852108

[68] Canchaya C, Proux C, Fournous G, Bruttin A, Brüssow H. 2003. Prophage genomics. Microbiol Mol Biol Rev 67:238–276, doi:10.1128/MMBR.67.2.238-276.2003 12794192 PMC156470

[69] Lood C, Boeckaerts D, Stock M, De Baets B, Lavigne R, van Noort V, Briers Y. 2022. Digital phagograms: predicting phage infectivity through a multilayer machine learning approach. Curr Opin Virol 52:174–181. doi:10.1016/j.coviro.2021.12.004 34952265

[70] Bajiya N, Dhall A, Aggarwal S, Raghava GPS. 2023. Advances in the field of phage-based therapy with special emphasis on computational resources. Brief Bioinform 24:bbac574. doi:10.1093/bib/bbac574 36575815

[71] Zünd M, Ruscheweyh H-J, Field CM, Meyer N, Cuenca M, Hoces D, Hardt W-D, Sunagawa S. 2021. High throughput sequencing provides exact genomic locations of inducible prophages and accurate phage-to-host ratios in gut microbial strains. Microbiome 9:77. doi:10.1186/s40168-021-01033-w 33781335 PMC8008629

[72] Shang J, Sun Y. 2022. CHERRY: a computational metHod for accuratE pRediction of virus–pRokarYotic interactions using a graph encoder–decoder model. Brief Bioinform 23:bbac182. doi:10.1093/bib/bbac182 35595715 PMC9487644

[73] Lu C, Zhang Z, Cai Z, Zhu Z, Qiu Y, Wu A, Jiang T, Zheng H, Peng Y. 2021. Prokaryotic virus host predictor: a Gaussian model for host prediction of prokaryotic viruses in metagenomics. BMC Biol 19:5. doi:10.1186/s12915-020-00938-6 33441133 PMC7807511

[74] Wang RH, Yang S, Liu Z, Zhang Y, Wang X, Xu Z, Wang J, Li SC. 2024. PhageScope: a well-annotated bacteriophage database with automatic analyses and visualizations. Nucleic Acids Res 52:D756–D761. doi:10.1093/nar/gkad979 37904614 PMC10767790

[75] Nayfach S, Camargo AP, Schulz F, Eloe-Fadrosh E, Roux S, Kyrpides NC. 2021. CheckV assesses the quality and completeness of metagenome-assembled viral genomes. Nat Biotechnol 39:578–585. doi:10.1038/s41587-020-00774-7 33349699 PMC8116208

[76] Xie J, Chen Y, Cai G, Cai R, Hu Z, Wang H. 2023. Tree visualization by one table (tvBOT): a web application for visualizing, modifying and annotating phylogenetic trees. Nucleic Acids Res 51:W587–W592. doi:10.1093/nar/gkad359 37144476 PMC10320113

[77] Liu B, Zheng D, Zhou S, Chen L, Yang J. 2022. VFDB 2022: a general classification scheme for bacterial virulence factors. Nucleic Acids Res 50:D912–D917. doi:10.1093/nar/gkab1107 34850947 PMC8728188

[78] Alcock BP, Raphenya AR, Lau TTY, Tsang KK, Bouchard M, Edalatmand A, Huynh W, Nguyen A-LV, Cheng AA, Liu S, et al.. 2020. CARD 2020: antibiotic resistome surveillance with the comprehensive antibiotic resistance database. Nucleic Acids Res 48:D517–D525. doi:10.1093/nar/gkz935 31665441 PMC7145624

[79] Brown CL, Mullet J, Hindi F, Stoll JE, Gupta S, Choi M, Keenum I, Vikesland P, Pruden A, Zhang L. 2022. mobileOG-db: a manually curated database of protein families mediating the life cycle of bacterial mobile genetic elements. Appl Environ Microbiol 88:e00991-22. doi:10.1128/aem.00991-22 36036594 PMC9499024

[80] Zheng J, Ge Q, Yan Y, Zhang X, Huang L, Yin Y. 2023. dbCAN3: automated carbohydrate-active enzyme and substrate annotation. Nucleic Acids Res 51:W115–W121. doi:10.1093/nar/gkad328 37125649 PMC10320055

[81] Yang M, Chen T, Liu YX, Huang L. 2024. Visualizing set relationships: EVenn’s comprehensive approach to Venn diagrams. Imeta 3:e184. doi:10.1002/imt2.184 38898979 PMC11183158

[82] Shaffer M, Borton MA, McGivern BB, Zayed AA, La Rosa SL, Solden LM, Liu P, Narrowe AB, Rodríguez-Ramos J, Bolduc B, Gazitúa MC, Daly RA, Smith GJ, Vik DR, Pope PB, Sullivan MB, Roux S, Wrighton KC. 2020. DRAM for distilling microbial metabolism to automate the curation of microbiome function. Nucleic Acids Res 48:8883–8900. doi:10.1093/nar/gkaa621 32766782 PMC7498326

[83] Li W, Cowley A, Uludag M, Gur T, McWilliam H, Squizzato S, Park YM, Buso N, Lopez R. 2015. The EMBL-EBI bioinformatics web and programmatic tools framework. Nucleic Acids Res 43:W580–W584. doi:10.1093/nar/gkv279 25845596 PMC4489272

[84] Chowdhury R, Bouatta N, Biswas S, Floristean C, Kharkar A, Roy K, Rochereau C, Ahdritz G, Zhang J, Church GM, Sorger PK, AlQuraishi M. 2022. Single-sequence protein structure prediction using a language model and deep learning. Nat Biotechnol 40:1617–1623. doi:10.1038/s41587-022-01432-w 36192636 PMC10440047

[85] Lu XJ. 2020. DSSR-enabled innovative schematics of 3D nucleic acid structures with PyMOL. Nucleic Acids Res 48:e74. doi:10.1093/nar/gkaa426 32442277 PMC7367123

[86] Shang J, Peng C, Liao H, Tang X, Sun Y. 2023. PhaBOX: a web server for identifying and characterizing phage contigs in metagenomic data. Bioinform Adv 3:vbad101. doi:10.1093/bioadv/vbad101 37641717 PMC10460485

[87] Otasek D, Morris JH, Bouças J, Pico AR, Demchak B. 2019. Cytoscape automation: empowering workflow-based network analysis. Genome Biol 20:185. doi:10.1186/s13059-019-1758-4 31477170 PMC6717989

