# Supplementary figures and images for: Unveiling the role of phages in shaping the periodontal microbial ecosystem
Source: mSystems. 2025 Mar 28;10(4):e00201-25. doi: 10.1128/msystems.00201-25 (PMC12013270; doi:10.1128/msystems.00201-25)

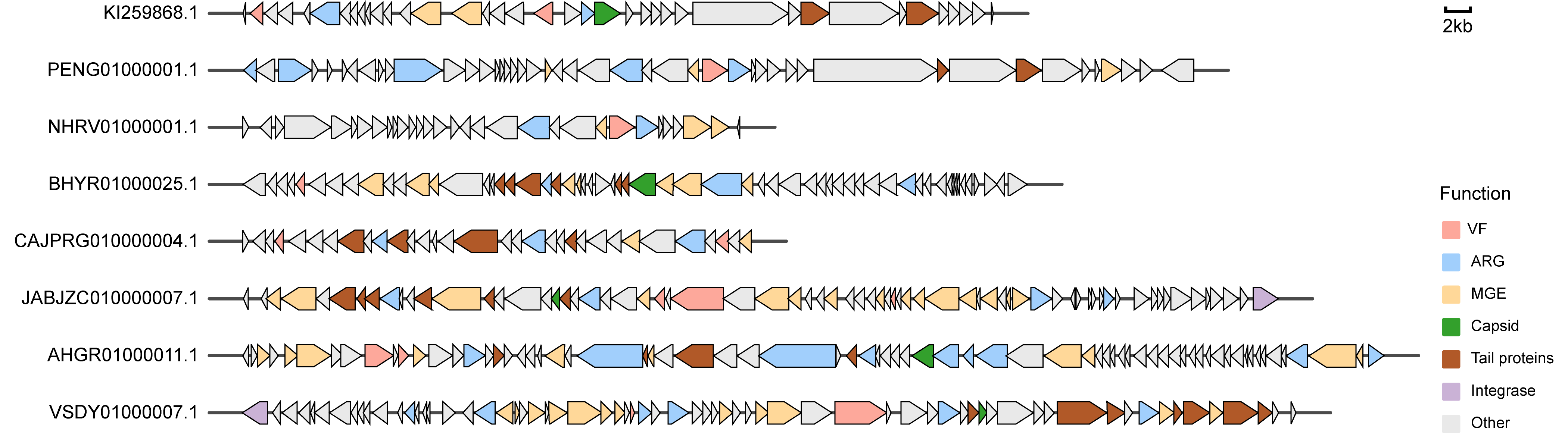

Supplement: Fig. S1 — Whole-genome maps of representative prophages containing VF, ARG, MGE, and prophage hallmark genes. [file msystems.00201-25-s0001.tif]

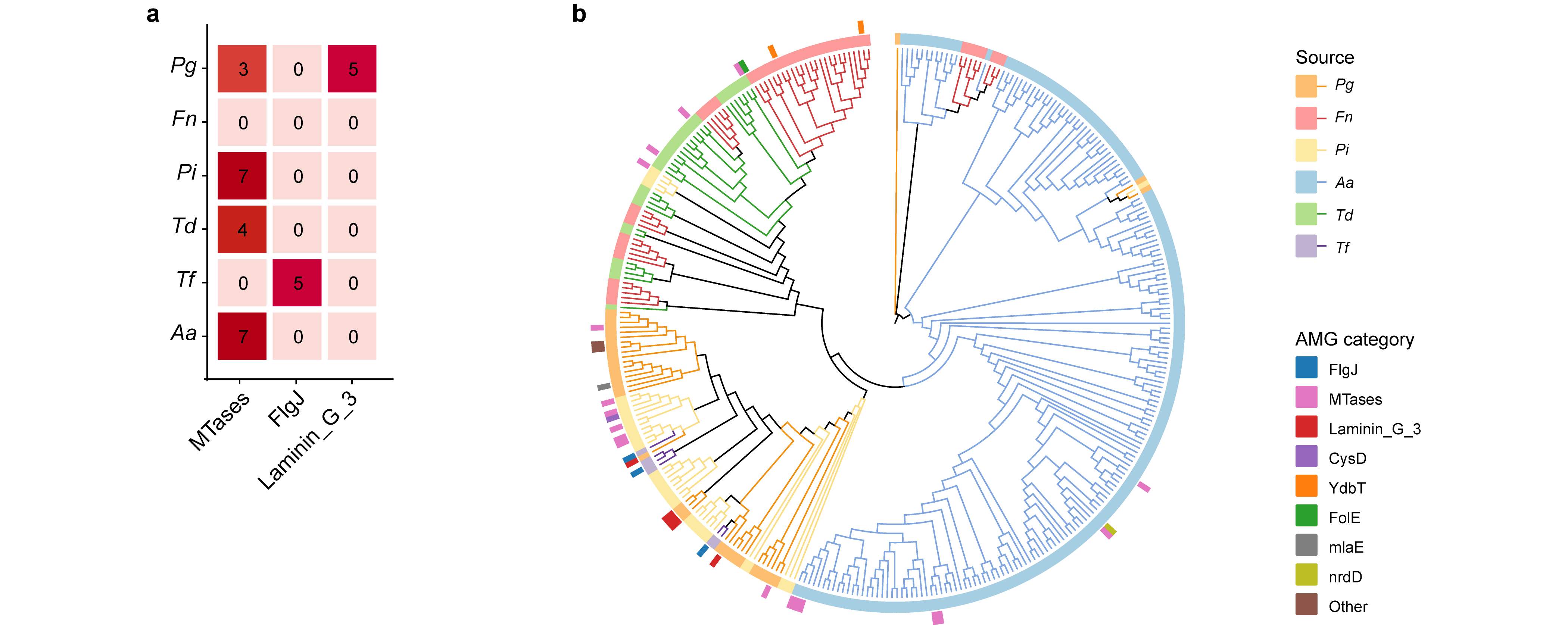

Supplement: Fig. S2 — Characterization of auxiliary metabolic genes in prophages. [file msystems.00201-25-s0002.tif]

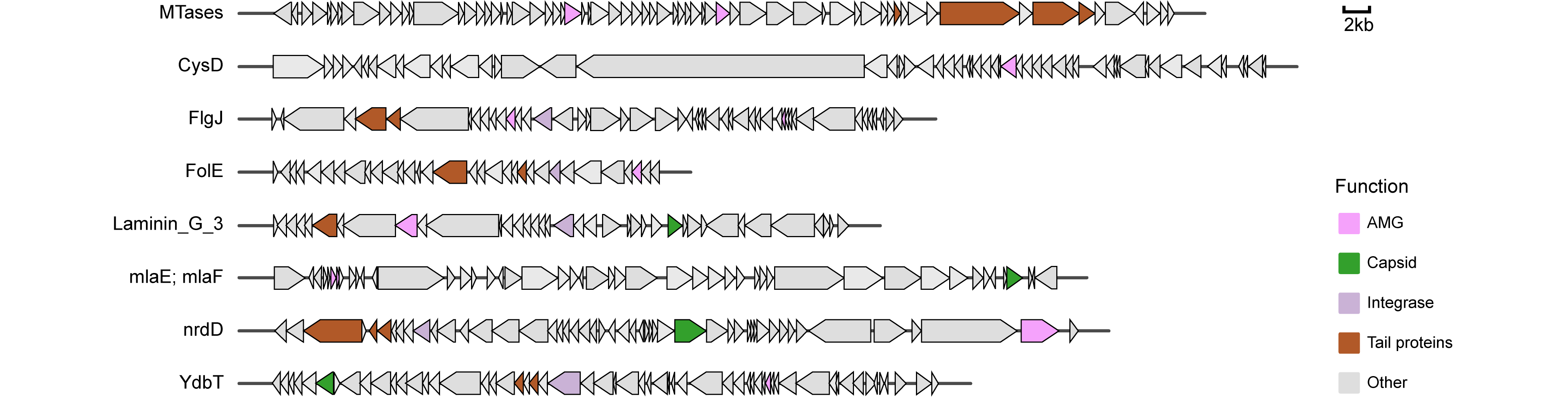

Supplement: Fig. S3 — Whole-genome maps of representative prophages carrying the auxiliary metabolic genes. [file msystems.00201-25-s0003.tif]

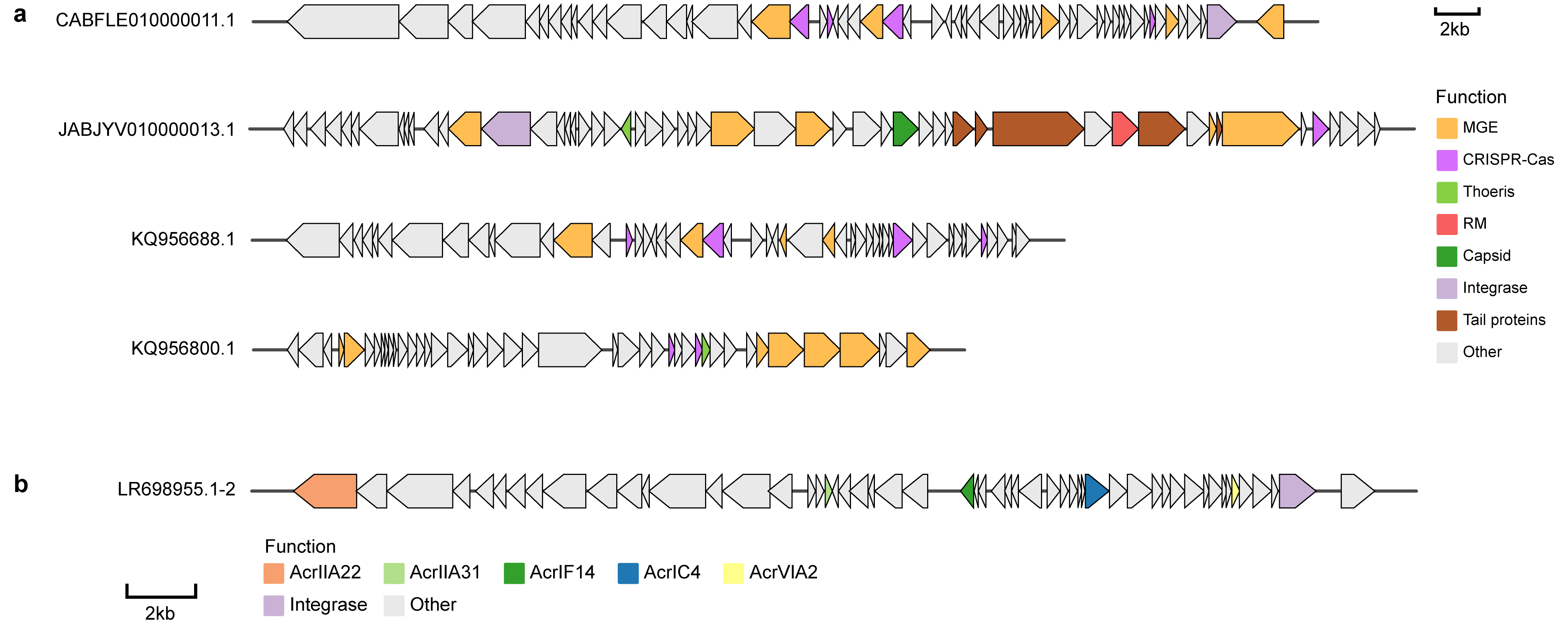

Supplement: Fig. S4 — Whole-genome maps of prophages carrying the anti-prokaryotic defense system genes. [file msystems.00201-25-s0004.tif]
