# Supplementary material for: Unveiling the role of phages in shaping the periodontal microbial ecosystem
Source: mSystems. 2025 Mar 28;10(4):e00201-25. doi: 10.1128/msystems.00201-25 (PMC12013270; doi:10.1128/msystems.00201-25)
Supplement: Legends — for Fig. S1 to S4. [file msystems.00201-25-s0005.docx]

**Supplementary materials**

Unveiling the role of phages in shaping the periodontal microbial ecosystem

Fangfang Yao^1#^, Jiajun He^2#^, Raphael Nyaruaba^3^, Hongping Wei^3*^, Yuhong Li^1*^

^1^State Key Laboratory of Oral & Maxillofacial Reconstruction and Regeneration, Key Laboratory of Oral Biomedicine Ministry of Education, Hubei Key Laboratory of Stomatology, School & Hospital of Stomatology, Wuhan University, Wuhan 430079, China

^2^CAS-Key Laboratory of Synthetic Biology, CAS Center for Excellence in Molecular Plant Sciences, Institute of Plant Physiology and Ecology, Chinese Academy of Sciences, Shanghai 200032, China; University of Chinese Academy of Sciences, Beijing 100039, China.

^3^Key Laboratory of Virology and Biosafety, Wuhan Institute of Virology, Chinese Academy of Sciences, Wuhan 430071, China.

For correspondence: *Hongping Wei: E-mail: hpwei@wh.iov.cn; *Yuhong Li: E-mail: 1004809372@whu.edu.cn.

^#^These authors contributed equally to this work

**Figure S1. Whole genome maps of representative prophages containing VF, ARG, MGE and prophage hallmark genes.**

Genes were marked by different colors to illustrate VF genes (red), ARGs (blue), MGEs (orange), Capsid genes (dark green), Tail protein genes (brown), Integrase genes (dark purple), and other phage genes (grey). Scale bar, 2kb.

**Figure S2. Characterization of auxiliary metabolic genes in prophages.**

(a) The number of AMGs in prophages from different hosts. The abbreviations represent the following bacterial strains: *F. nucleatum* (Fn), *P. gingivalis* (Pg), *P. intermedia* (Pi), *T. forsythia* (Tf), *T. denticola* (Td) and *A. actinomycetemcomitans* (Aa). (b) A phylogenetic tree of prophage is presented.The inner blocks indicate the host bacteria of the prophage. The outer blocks represent the AMGs in prophage.

**Figure S3. Whole genome maps of representative prophages carrying the auxiliary metabolic genes.**

Genes were marked by different colors to AMGs (cerise), Capsid genes (dark green), Tail protein genes (brown), Integrase genes (dark purple), and other phage protein genes (grey). Scale bar, 2kb.

**Figure S4. Whole genome maps of prophages carrying the anti-prokaryotic defense system genes.**

1. Whole genome maps of prophages carrying the anti-prokaryotic defense system genes. Genes of these anti-defense systems were depicted in different colors, such as CRISPR-Cas (light purple), Thoeris (light green), RM (red). Meanwhile, other genes are also colored accordingly: MGEs (orange), Capsid genes (dark green), Tail protein genes (brown), Integrase genes (dark purple), and other phage genes (gray). (b) Whole genome maps of prophage carrying the anti-CRISPR genes. Genes were marked by different colors to AcrIIA22 (orange), AcrIIA31 (light green), AcrIF14 (dark green), AcrIC4 (blue), AcrVIA2 (yellow). Integrase genes (dark purple), and other phage protein genes (grey). Scale bar, 2kb.
